# A novel empirical classification method for weak rock slope stability analysis

**DOI:** 10.1038/s41598-022-19246-w

**Published:** 2022-08-30

**Authors:** Mohammad Azarafza, Masoud Hajialilue Bonab, Reza Derakhshani

**Affiliations:** 1 Department of Civil Engineering, University of Tabriz, Tabriz, Iran; 2 Department of Earth Sciences, Utrecht University, Utrecht, The Netherlands

**Keywords:** Engineering, Civil engineering

## Abstract

This study presents a novel empirical classification system for stability analysis of rock slopes in weak rock based on their geotechnical and geological properties. For this purpose, consideration is given to the marly rock slopes, which include three main groups of weak rock (lime marlstone, marlstone, and marly limestone). The 40 different slopes located in the South Pars special zone (Assalouyeh), southwest of Iran, are targeted in classification. To prepare comprehensive graphical stability charts for weak rocks, extensive field surveys, sampling, geotechnical laboratory tests, and ground measurements are conducted in slope sites. Using the findings of the study, empirical stability charts for slopes composed of weak materials were developed. The charts are associated with geotechnical indexes, geo-units’ weathering impact, and in-situ stress conditions. Using these graphical charts assists in investigating the stability condition of rock slopes and estimating the geotechnical characteristics of clay-based weak rocks such as marlstones.

## Introduction

Slope stability is the extensive description of rock and/or soil mass displacements, movements, and failures^1^ under different triggering factors which lead to slope instabilities at various scales^2^. The instabilities are mostly controlled by certain conditions^3–5^ that can be related to the slope geometry, discontinuity network, geo-materials of host mass, geological structures, static and/or dynamic loading, and the critical slip surface developments^6^. There are different classifications to indicate the slope conditions during stability analysis. From a slip surface point of view, the planar, circular, non-circular (general), and composite forms of slip surface are identified in slope instabilities, which become much more complicated in three-dimensions^7–10^. Regarding the slope failure mechanism, a wide range of failures such as the wedge, planar, rotational, and toppling failures, composite slips, and special cases can be expressed^11,12^. Due to such extensive types of mass movements in slopes, there are various procedures developed and applied^13^. So far, various stability assessment techniques have been developed which include a range of simple evaluations, planar failure, limit state criteria, limit equilibrium analysis, empirical techniques, numerical methods, hybrid and high-order approaches which are implemented in two- and/or three-dimensions^14–16^. Each of these procedures is operated with specific requirements and computational assumptions. In the meantime, the empirical techniques can be considered as the basics of the other procedures which are founded on technical experiences over many years and provide a quick analysis with minimum assumptions^17^.

Geotechnical practitioners have developed empirical methods for quickly quantifying and making decisions about stabilizations^18^. Most of the works on empirical classification for rock or soil slopes rely on engineering experiences; therefore, expert knowledge of slope mass conditions is required^19,20^. The researchers developed various classifications (mostly for rock masses) to quantify the mass condition and recommend stabilizations to correct the mass instabilities. Terzaghi^21^ and Ritter^22^ are some of the first scholars who work on empirical classification systems for geo-materials stabilizations. Subsequently, researchers like Lauffer^23^, Barton et al.^24^, Cecil^25^, Selby^26^, Deere and Deere^27^, Bieniawski^28^, Chen^29^, Singh and Geol^30^, Hack et al.^31^, Romana et al.^32^, and Marinos et al.^33^ provide the various quantifications system for different civil and mining engineering purposes. Over time, these classifications are modified and corrected, which leads to more accurate categorizations^34^. There are many classification systems and stability charts are developed based on the presented classification systems, several of which are specifically used for rock slopes^30^. Most of these classification systems are focused on intact material strength conditions (like uniaxial compressive strength, UCS) and slope mass geometrical properties^35^. The geological condition plays a key role in the stability of the slope. These factors are not considered properly in some of the classifications. The geological conditions directly control the phenomena that suppress the slope’s durability, such as weathering^36^. The presented article attempted to introduce a novel classification system for sedimentary rock slopes regarding stability conditions, rock failure, and geotechnical properties. In this regard, various aspects of the geological condition, slope mass features, and geotechnical properties were considered to develop stability charts.

## Studied case

**Figure 1:**
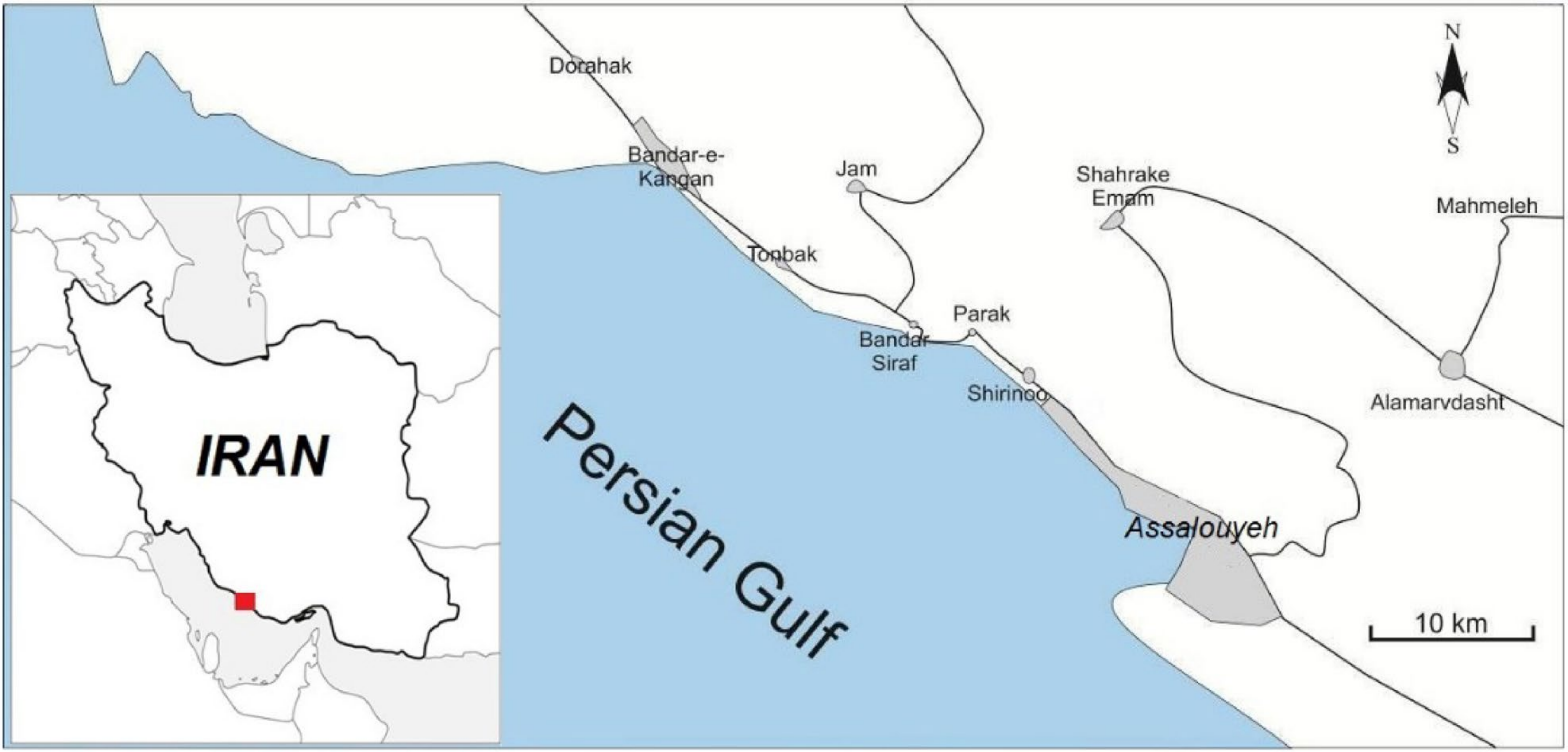
Location of the South Pars. The map is created using CorelDraw version 2018 (https://www.coreldraw.com/en/?link=wm).

The South Pars region is a narrow area located in Assalouyeh, Bushehr province, southwest of Iran (Fig. 1). South Pars is known as Iran's largest refinery site with hydrocarbon facility centers. The geo-structural and topographical variations indicate that the South Pars has a complex geological and tectonic situation. The collision of the Arabian and Central Iranian plates, which resulted in the NW–SE striking the Zagros mountain range^37^ with active folds, faults^38^, and running tectonic deformation^39^, is the main cause of this geological situation. This region has an approximate area of over 10,000 hectares, which is covered by different geological units ranging from the late Neo-Proterozoic to recent alluviums. In the core of the Assalouyeh anticline, formations older than the Cenozoic (Asmari formation) are exposed, while the SPZ region is mostly covered by post-Asmari (Eocene–Oligocene) Mishan, Aghajari, and Bakhtiari formations and Quaternary sediments. The studied units are related to the marly materials which represent parts of the Mishan formation (molasses, carbonate, and siliciclastic facies deposited in a carbonate rimmed shelf and of gray marl and marlstone with clay layers, olive-green to gray and sometimes red marls), the Aghajari formation (fine, medium and coarse-grained sediments, usually interpreted as channel deposits and alternating gray to brown calcareous sandstone, gray, dark green and pink to red marls with veins of gypsum, gray marls, and green siltstone) with an age attributed to Miocene and Pliocene and alluvial deposits.

## Geo-engineering characteristics in slope stability

**Figure 2:**
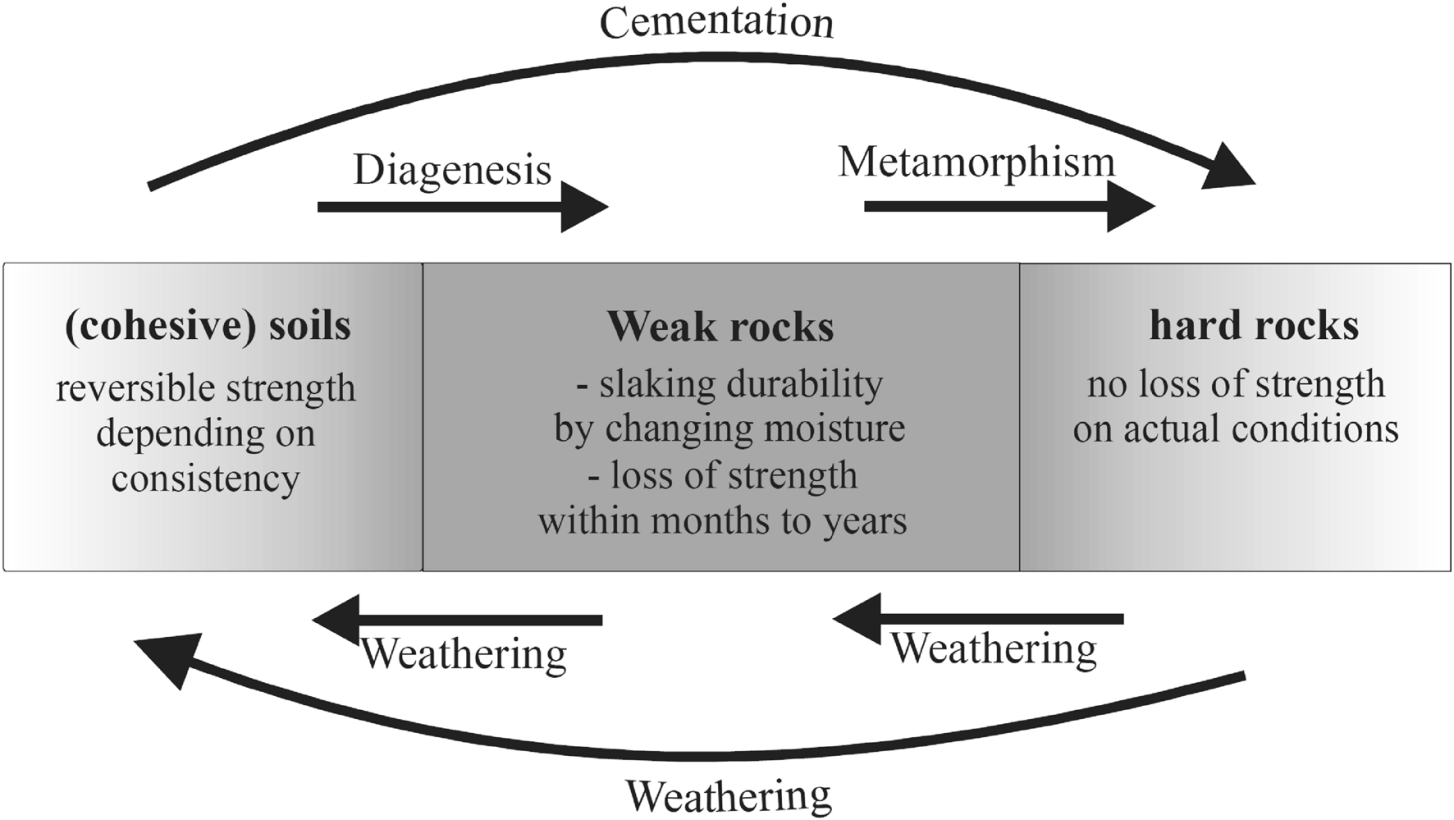
Geological process in earth surface^38^.

**Figure 3:**
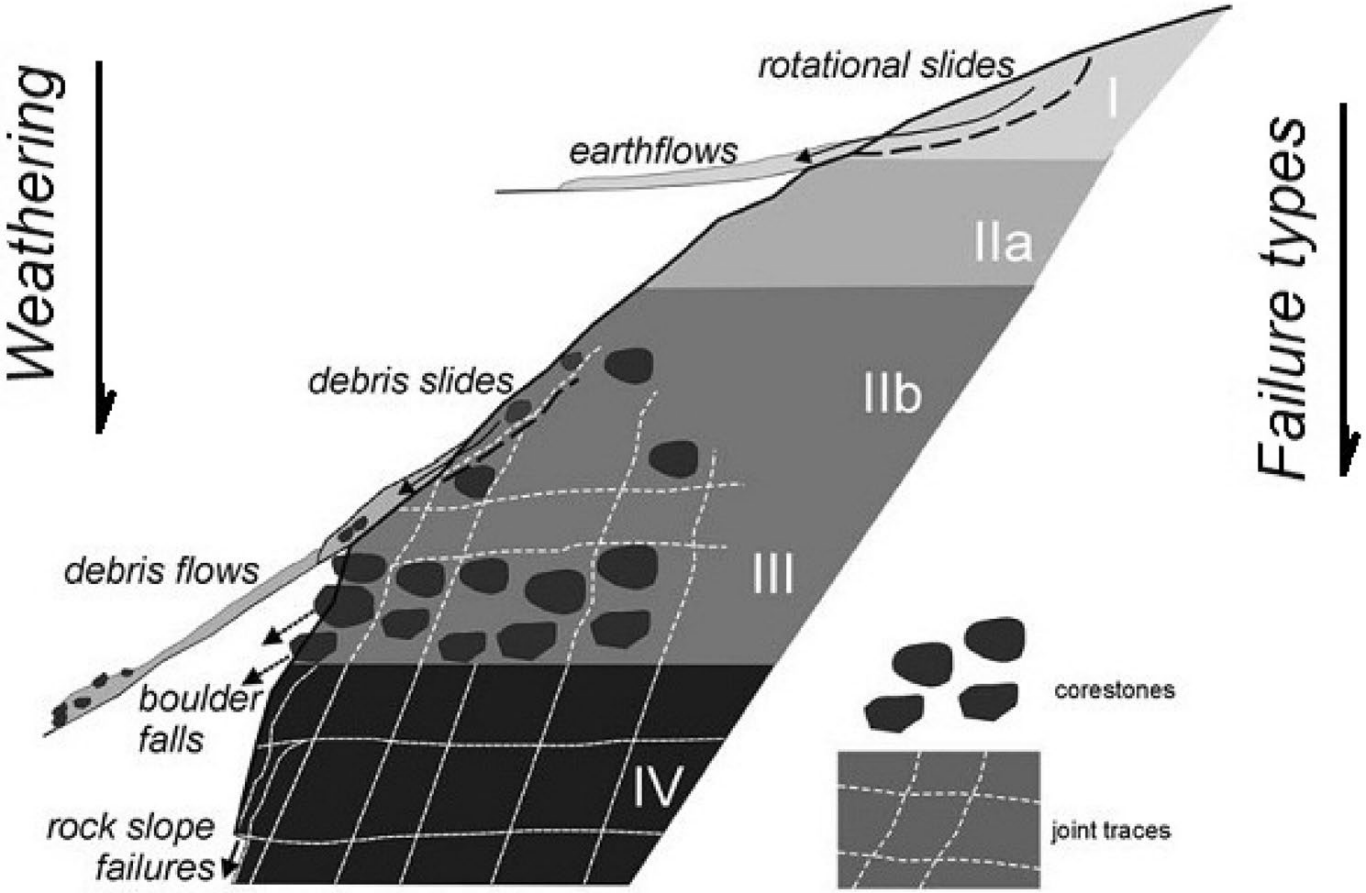
Weathering process impact on slope instabilities^39^.

Various engineering-geological parameters are involved in the stability of the slopes. Among these, we can mention the geometry, material and environmental properties of the slope masses are very important^40^. Nickman et al.^35^ described the geological process (weathering-cementation) in geological units and mentioned it is a continuous time-dependent process. Figure 2 shows the weathering-cementation process on the earth's surface (rock materials). Calcaterra and Parise^41^ stated that depending on the different weathering stages, the various slope movements (instabilities) occur by different geo-structural involvement which is shown in Fig. 3. As shown in this figure, the weathering process occurs in four stages, culminating in flows or rotation failure (massive movements) in soil or debris alluvial. This instability covers groups *I* and *IIa*. Debris can occur with sliding on a planar or non-circular failure surface in group *IIb*. With the transformation of geological units into regular networks of rock blocks, failure events take on a more consistent structure, and various forms of failure, like rock-fall, slide, wedge, planar, toppling, and composite failure appear. Groups *III* and *IV* are responsible for structured slope failures in geo-materials. During the weathering process, rock masses change from hard to weak and weak to soil. This transformation affects the failure mechanisms regarding the geological condition of slope mass^12^, which leads to different types of instabilities in slope mass^1^. Thus, it can be stated that geological processes like weathering can change the type, mechanism, and scale of the instabilities in rock slopes. Figure 2 indicates the variation of failure types on a slope with weak materials. With regard to the increasing weathering degrees in weak rocks like marls, the slope instability becomes mass movement from structural failures. In fact, with increasing weathering, the joint system is destroyed, the rock structure is destroyed, the result is soil. In rock masses, structured failures are generally observed in which weathering is low and joints are responsible for instabilities (Class *I*); but in the soil materials, the sliding surface passes through the slope mass (Class *IV*).

The change in the nature of the geo-materials in a slope is the cause of instability, which has led geo-engineers to consider such geological behavior as one of the effective elements in the main failure of slopes^42^. Geometric and geo-material changes in slopes cause instability, which can be directly controlled by the angle of repose or critical angle of repose in slopes^43^. Mehta and Barker^42^ mentioned that the critical angle of repose is commonly equal to the tangent of the slope angle (*β*) which is estimated by the experimental survey from various fields. Table 1 provides information about some geomaterials' critical angle of repose.

1 $$ \mu_{s} \approx \tan \beta \,\,\,\,\,\,\,\,\,\,\,\,\,\,\,\,\,\,\,\,\,\left\{ \begin{gathered} {\text{Slope}}\,{\text{is}}\,{\text{stable}}\,\,{\text{if}}\,\,\beta { < }\varphi \hfill \\ {\text{Slope}}\,{\text{is}}\,{\text{unstable}}\,\,{\text{if}}\,\,\beta { > }\varphi \hfill \\ \end{gathered} \right. $$

where *μ*_*s*_ is the critical angle of repose, *β* is the slope angle, and *ϕ* is the friction angle of the materials. Using the geotechnical characteristics, field survey, and geological condition of the slope mass, it is possible to provide a link to describe various stability states of the slope.

**Table 1:** A estimated critical angle of repose for several geo-materials^41^.

Materials	μ_s_ (^o^)	Materials	μ_s_ (^o^)	Materials	μ_s_ (^o^)
Asphalt	30–45	Soil (general)	30–45	Gravel	25–30
Dry clay	25–40	Granite	35–40	Dry sand	34
Wet clay	15	Gravel (coarse)	45	Wet sand	45
Snow	38	Chalk	45	Ash	40

Viles^43^ stated that the weathering rate-limiting nature of rock slopes is a result of different factors, especially climate changes, rainfall, temperature, geomorphology, and geology, which directly affect the ratio of strength to stress. So, the weathering could be linked to geo-material strength, while the weathering conditions triggered the slope to slide by providing weakness in the rock mass and stresses^44^. In this regard, it would be quite logical to state that weathering has a direct effect on the geotechnical properties of the rock mass. Hall et al.^45^ mentioned that weathering is an in-situ breakdown of rocks with effects on their durability, chain of compounds, and stiffness. Ollier^46^ provides evidence that large-scale weathering can trigger landslides. Viles^43^ provides the table of variation where weathering leads to slope instabilities and prepares a link between the in-situ stress field and the slope’s shallow and deep failures^47–49^. The authors indicate the geological units can reshape the sliding surface in slope mass. So, it can result in the weathering providing complexity in the failure mechanism, slip surface, and scale of the mass movements. Miščević and Vlastelica^36^ by conducting an experimental survey on clayey-dominant rocks stated that the weathering affected geo-materials engineering properties by reducing shear strength, increasing porosity, and the structural breakup of materials. So, it has an impact on various aspects of geoengineering features. Based on this fact, an attempt has been made to investigate these dimensions of impact in this study and, subsequently, their effects on slope stability.

## Analysis method

The presented study attempted to provide an empirical solution for rock slopes faced with various types of instabilities regardless of the type of failure mechanism. As is known, the regular classification systems are developed based on the geometry and discontinuity network of the slope mass, which is mostly lighted by the weathering impact on rock materials and associated with corrections in weak rocks. The presented method uses the detailed classification for weak rocks, especially marls, which are mainly not considered in existing classifications. The proposed method was established on a comprehensive field survey of 40 different slopes located in the South Pars special zone (Assalouyeh), southwest of Iran. For estimation of geotechnical characteristics, various geotechnical experiments were conducted on weak rock specimens (40 samples were taken from the slopes). Geologically, slopes consist of marlstones, limey marls, and marly lime geo-units. During the field survey, the appearance conditions of slopes along with their geomechanical properties have been harvested and recorded. Geotechnical tests like *UCS* and direct-shear were used to estimate the geomechanical properties of the rock materials. After providing the relevant information about the slopes during the field survey, like slope angle (*β*), internal friction angle (*ϕ*), cohesion (*c*), shear strength (*τ*), and slope height (*H*), the parameters are used for stability assessment. Several empirical stability charts have been introduced to estimate the factor of safety (F.S) by using geotechnical and geological conditions. The weathering effect is important in the evaluation by using experimental impact factors regarding the degree of weathering. The utilized impact factors are presented in Table 2. These impact factors were derived from the field survey and the durability of the marly rocks in the area under study.

**Table 2:** The variation of impact factors in stability analysis of slopes based on the proposed method.

Class	Parameter				
	Excellent	Good	Fair	Weak	Very weak
Weathering	Fresh	Slight	Moderate	Deep	Structural
Impact factor	0.9–1.0	0.6–0.9	0.4–0.6	0.2–0.4	0.0–0.2
Support system	none	Light	Shelf required	Coupled	Heavy

During the field survey in South Pars, several sampling locations were selected, which covered about 40 different slopes. The topography and discontinuity conditions for each slope are recorded at the slope site, and sampling was performed to investigate the geo-engineering characteristics. Instructions for field studies, sampling, and testing are provided by the International Society for Rock Mechanics (ISRM) and American Society for Testing and Materials (ASTM) organizations, which are comprehensively described in geotechnical books. The samples, after being taken from the slope sites, were delivered to the geotechnical laboratory to estimate the geotechnical properties, stiffness, and strength parameters. The regular geotechnical tests were conducted on rock samples like *UCS*, direct-shear, and triaxial tests, which are standardized by ASTM. These tests are used to determine the geotechnical properties of intact rocks and should be modified into rock mass parameters by considering correction indices. To develop the stability charts, geotechnical tests were performed on taken samples from 40 different slope sites, and the results were used to provide the geo-engineering characteristics of marly materials. The samples were taken and isolated (to avoid the changes in water content of the sample), transferred into the laboratory, the samples were prepared, and tested to estimate the engineering properties. The geotechnical characteristics of the studied marls are presented in Table 3.

**Table 3:** Geotechnical characteristics for studied samples.

Parameter	Unit	Max	Min	Mean	St.Dv
Water content	%	12.37	1.78	7.07	7.48
Specific gravity (G_s_)	–	2.79	2.38	2.58	0.28
γ_t_	kN/m^3^	22.62	20.05	21.33	1.81
γ_d_	kN/m^3^	25.45	22.07	23.76	2.39
Porosity	%	23.38	8.77	16.07	10.33
Carbonate content	%	77	38	57.5	27.57
Cohesion (c)	kPa	320	97	208.5	157.6
Friction (ϕ)	degree	35	17	26	12.72

In order to provide the stability charts, the limit equilibrium analysis method (LEM) is used^2^. LEM is one of the basic analytical methods for slope stability analyses that is widely used in slope stability studies because of its simplicity, low complexity in the formulation, and less analysis time^4^. The LEMs based on massive analysis or slices investigate a possible slippery mass at the top of the assumed slip surface and the polyhedral force vector closure or incurring moments in equilibrium state, which are capable of being utilized in static and dynamic conditions for two-dimensional and three-dimensional space^5^. If these polyhedral forces are closed and all assumptions/requirements are provided, this implies that the mass is in equilibrium and that the analysis is valid. The non-closure of the polyhedral forces/moments indicates the lack of balance or lack of satisfaction of some effective parameters in it^4^. There are various LEM methods that are used by researchers for different purposes, which cover various failure types. Generally, the safety factor (F.S) in weak or weathered rock masses is not accurate to determine the slipping surface by one or several discontinuities or gaps, and this slipping surface passes through the path that has the least resistance. So, regarding the weathering degree (Fig. 3), the sliding surfaces are changed. Zhu et al.^16^ mentioned that in the two-dimensional stability analysis in the cross-sectional area, the slope is restricted by the ground surface (y = g [x]) and sliding surfaces (y = s [x]). Assuming that the coefficient is constant and equals the F.S for the entire sliding surface, the expansion of the slipping surface on the slider surface is determined as a function of the mass weight element in the static state W (x). Considering the validity of the Mohr–Coulomb failure criterion, it can be stated as:

2 $$ {\uptau }({\text{x}})\, = \,\frac{{\{ c(x) + [\sigma (x) - u(x)]\tan \varphi (x)\} }}{{{\text{F}}.{\text{S}}}} $$

3 $$  {\text{F}}.{\text{S}}  =  \frac{{\eta _{1} \int_{a}^{b} {\sigma _{0} \xi _{1} \psi r_{\tau } dx}  + \eta _{2} \int_{a}^{b} {\sigma _{0} \xi _{2} \psi r_{\tau } dx}  + \int_{a}^{b} {( - u\psi  + c)r_{\tau } dx} }}{{M_{c}  - \eta _{1} \int_{a}^{b} {\sigma _{0} \xi _{1} \psi r_{\tau } dx}  - \eta _{2} \int_{a}^{b} {\sigma _{0} \xi _{2} \psi r_{\tau } dx} }}  $$

**Figure 4:**
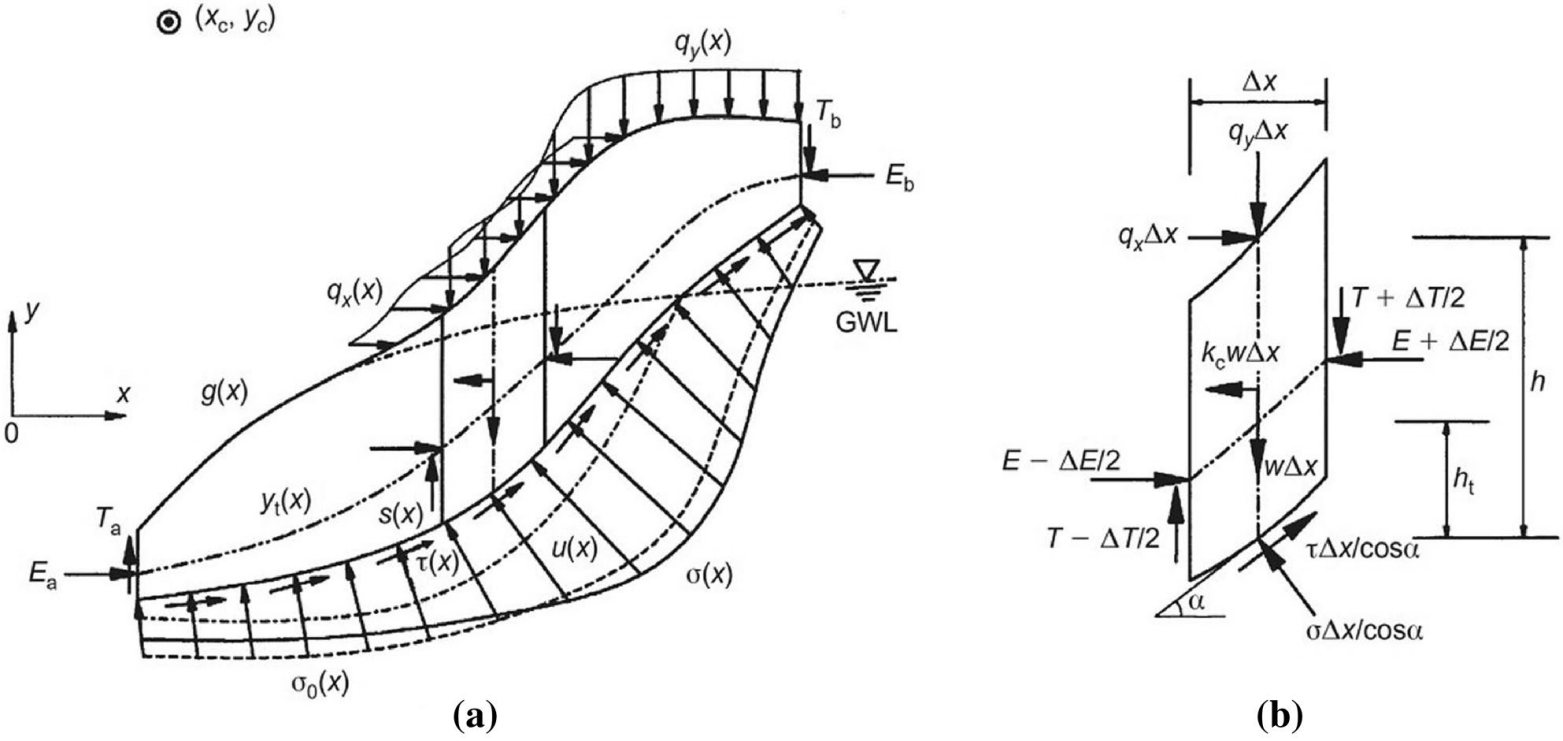
Generalized limit equilibrium-based slope stability analyses^16^.

The above equation can be generalized and extended to all limit equilibrium methods. Also, the requirements can be found in Fig. 4. These LEM relations can be expanded into the various types of failures with different sliding surfaces, from circular to composites. The presented study uses the Zhu method to develop stability charts for weak rocks.

## Results and discussion

**Figure 5:**
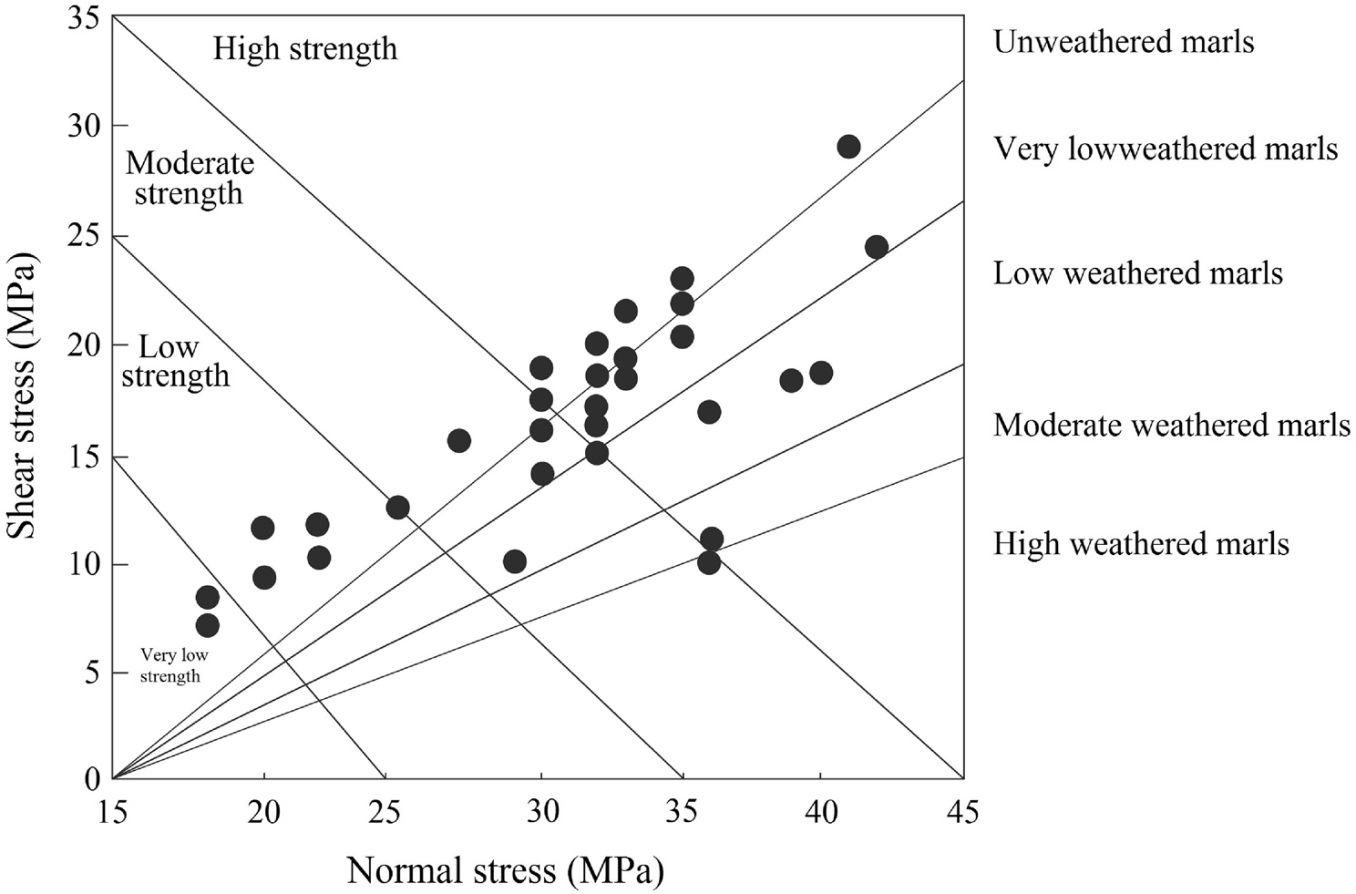
The in-situ stress variation of various marls with respect to the weathering condition.

**Figure 6:**
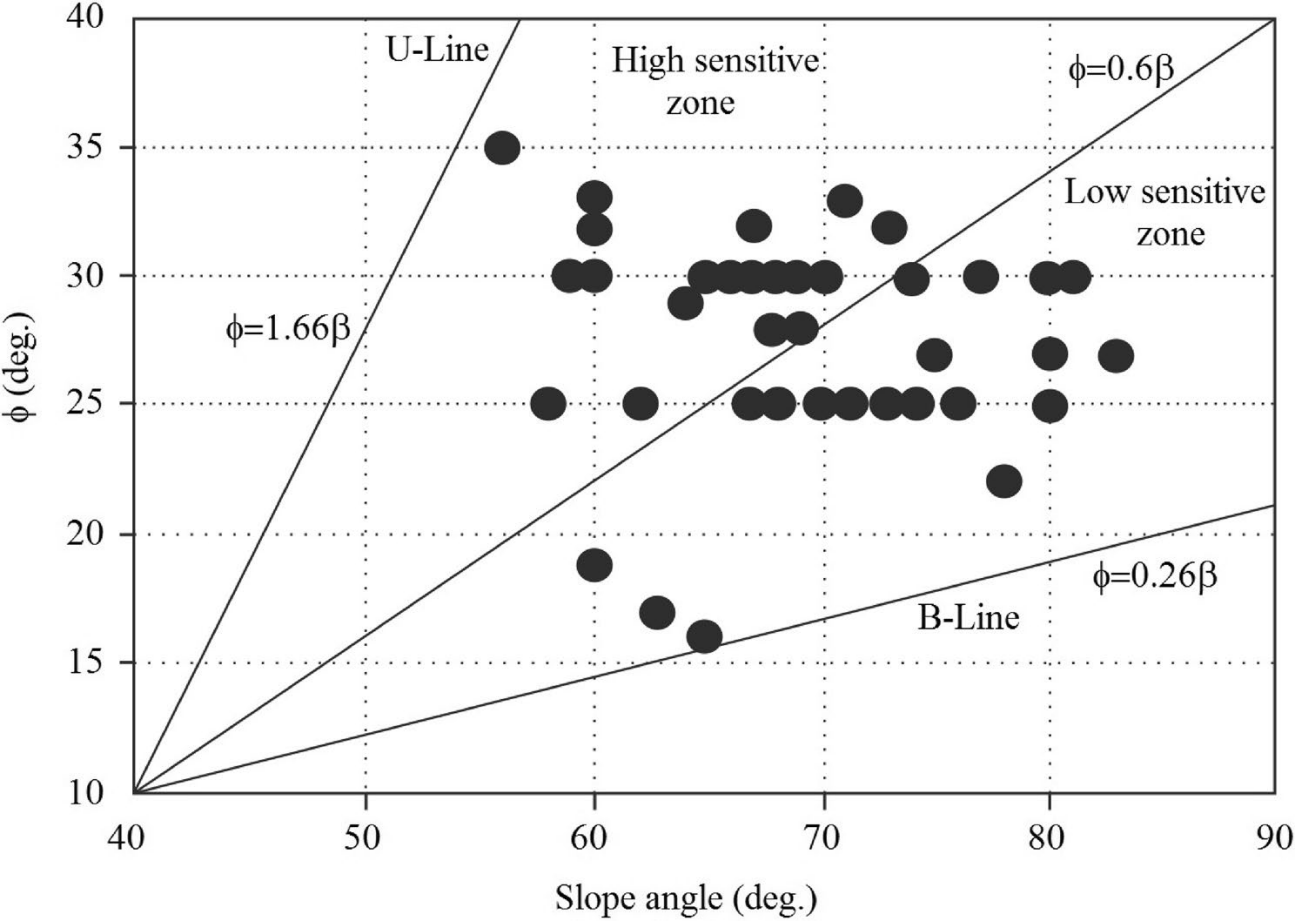
The sensitive condition of slope regarding weathering condition.

**Figure 7:**
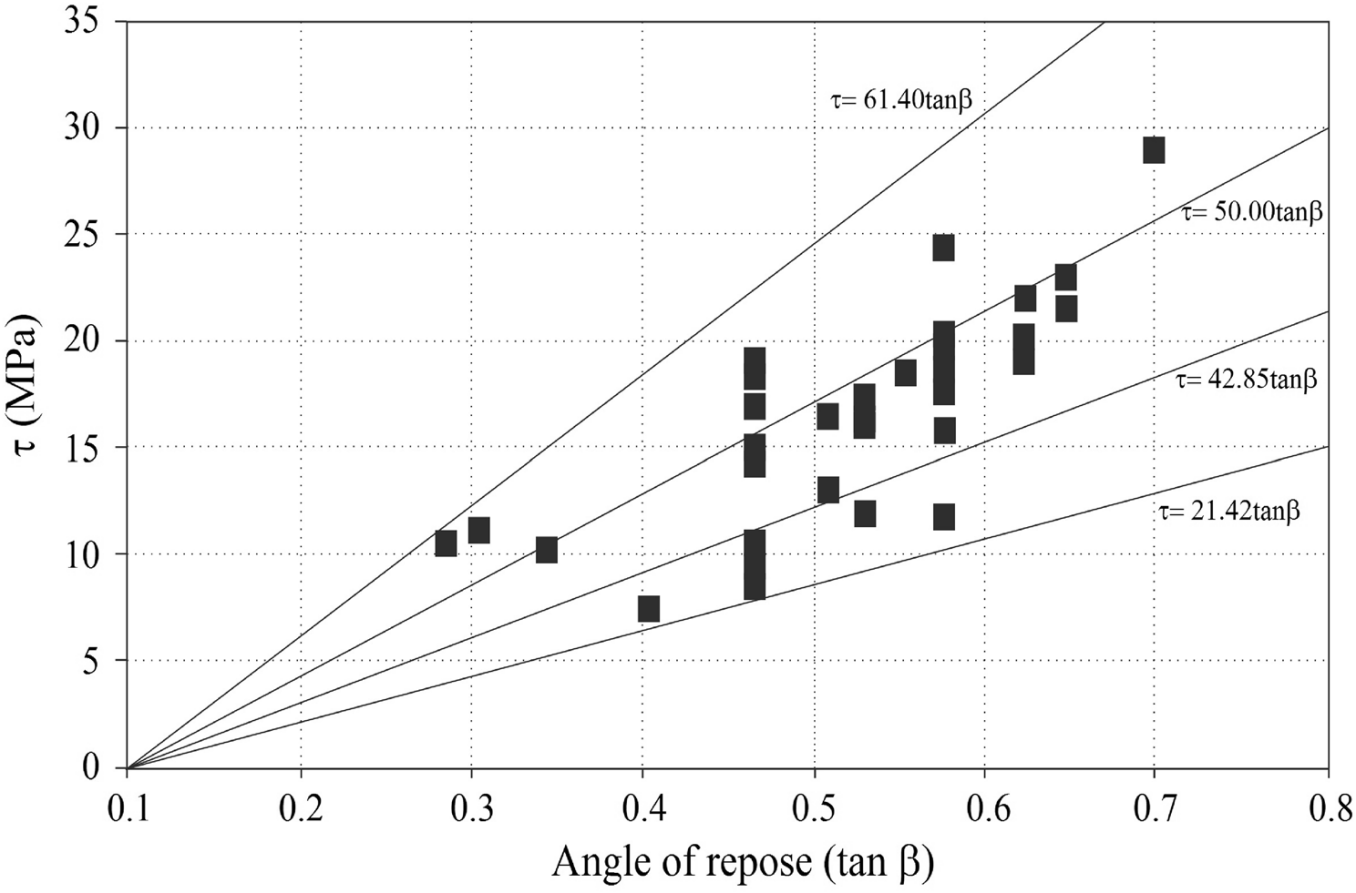
The shear strength variation of the slope based on the angle of repose regarding weathering condition.

**Figure 8:**
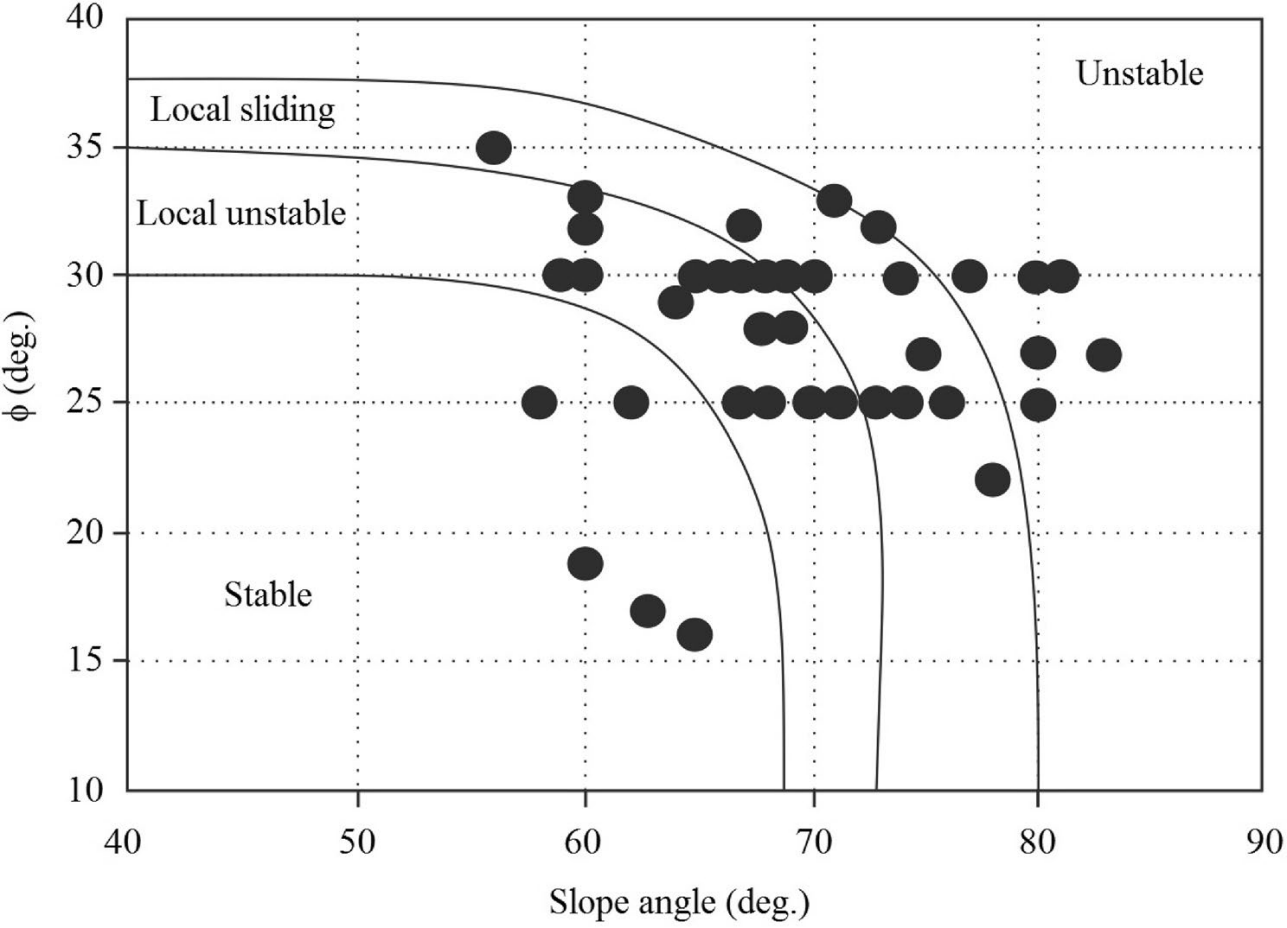
The stability chart for weak rocks.

Extensive ground and laboratory studies were conducted in an effort to develop stability charts for slopes, which led to the development of the proposed method. The charts help to understand the current stability situation of the slope. In addition, the charts facilitate estimating the geotechnical indexes in weak rocks (marls) with proper approximations. Figure 5 provides the first stability chart, which represents the normal and shear stress conditions. As illustrated in the figure, highly weathered marls are sheared at very low strength, while unweathered marls are sheared at high strength. Also, the shear and normal stress conditions were increased with the weathering degree. The weathered marls mostly indicated low shear strength compared to unweathered marls. But according to the field survey conducted in South Pars, there are several unweathered marl specimens that show low strength, which is mainly related to mineralogical conditions and clay particles in marls. The clay particles in marls lead to a reduction in the strength of rocks. So, this phenomenon has been responsible for the low strength in unweathered marls. Figures 6 and 7 illustrate the various stability charts regarding the geotechnical indexes and slope conditions, which describe the slope’s stability conditions. According to Fig. 6, it appears that the fresh marls to weathered marls show a relation between ϕ = 0.26 β to ϕ = 1.66 β. As this amount increases, the sensitivity of the instability in the slope also increases. The variation of the parameters is identified by U-line and B-line. Two upper and lower threshold limits can be considered an appropriate indicator for such parametric variations in slope mass. Figure 5 illustrates the information about ϕ versus β which provides the sensitivity of the slope regarding failures. The study indicated the ϕ between 1.66 β to 0.6 β shows a high risk of failure in marly slopes. Also, the ϕ between 0.6 β to 0.26 β shows a low risk of sliding. Regarding this figure, the lower values from B-line and upper values from U-lime don’t happen, which indicates the line of variations for the risk-ability of the marly slope to slide. Figure 7 provides a variation chart for *τ* and *μ*_*s*_ which is used to estimate the critical angle for slope failures in weak rocks. The results of the assessments show that the τ = 21.42 μs to τ = 61.4 μs from weathered to fresh marls. By considering the presented information, slopes are classified based on stability conditions as illustrated in Fig. 8. Figure 7 illustrates the variation in shear strength for marly slopes. The study appeared the τ increases with the angle of response in slops. The classification provided for various weathering degrees, which is τ and μ_s_, is increased by reducing the weathering level.

**Figure 9:**
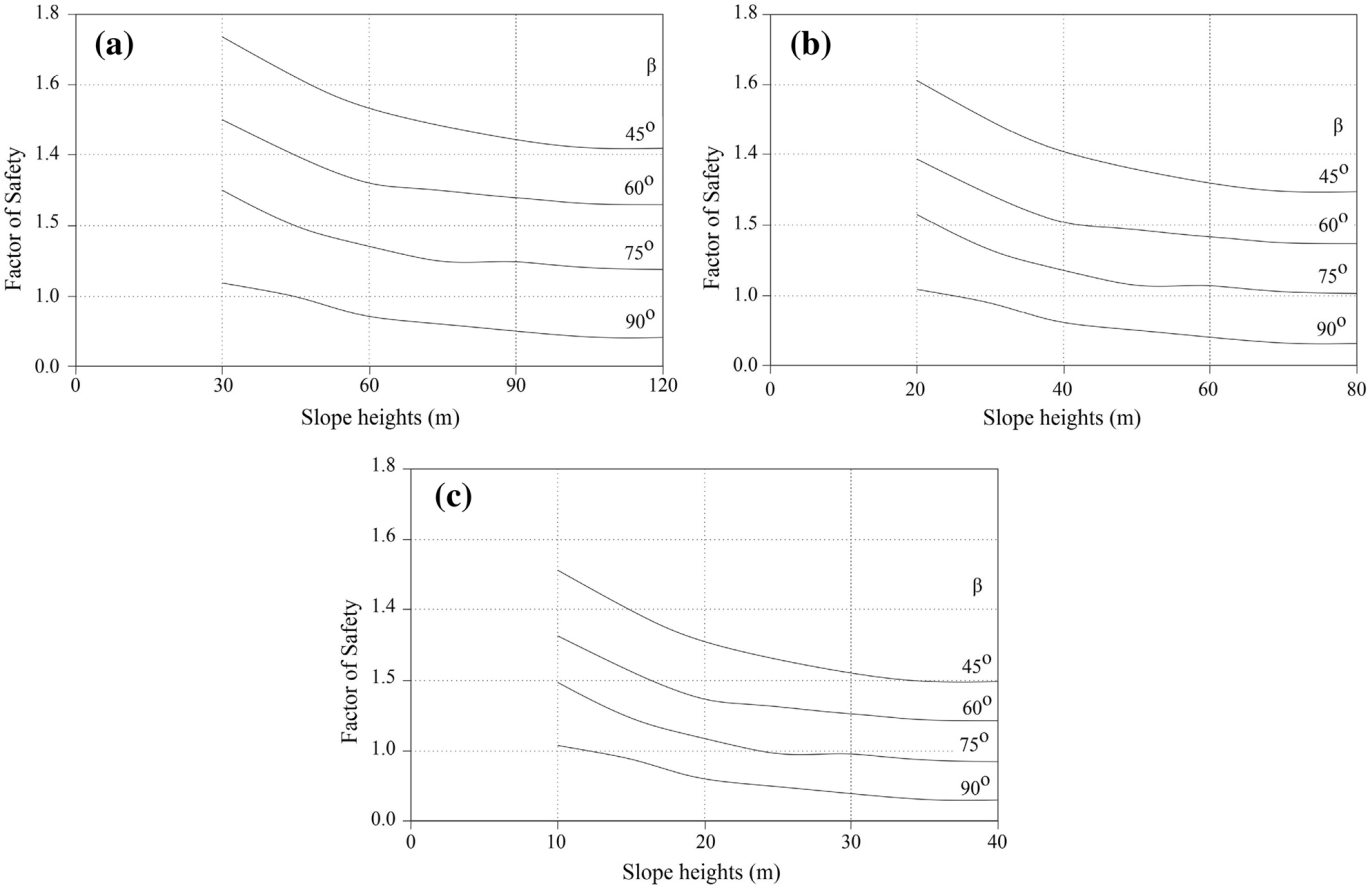
The stability chart for estimation of F.S in various marlstones: (**a**) limey marl, (**b**) marly lime, (**c**) marl.

**Figure 10:**
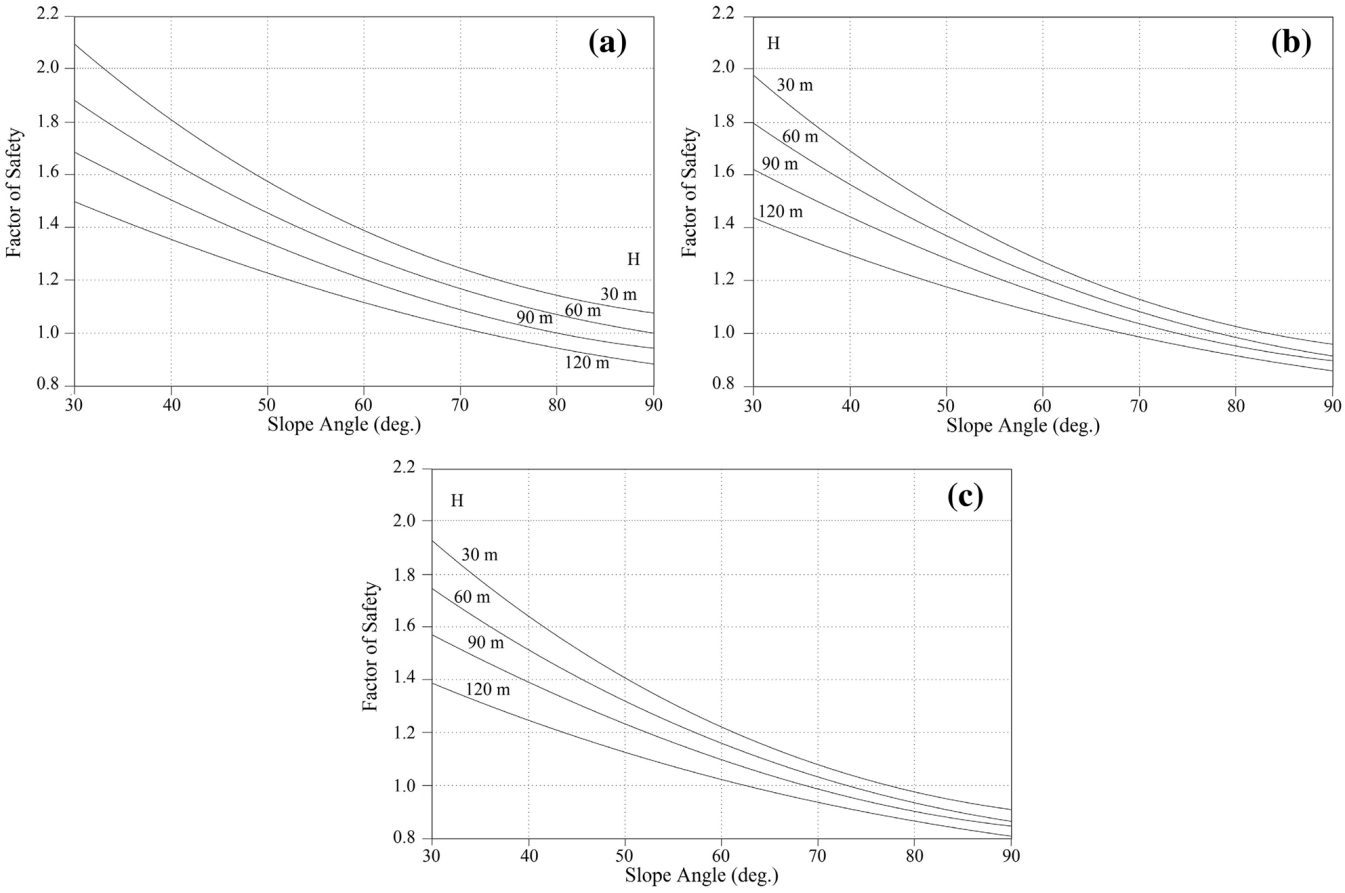
The stability chart for estimation of F.S in various marlstones: (**a**) limey marl, (**b**) marly lime, (**c**) marl.

By considering the relationship between *ϕ*, *β,* and slope instability, it is evident that marls undergo a variety of changes. This variation can be used to estimate the range of F.S based on the limit equilibrium assumption in slopes. The F.S variation in different slopes based on geometrical properties is illustrated in Figs. 9 and 10. These charts were utilized to estimate the F.S for various weak rocks subjected to distinct weathering conditions. Taking into account the preceding diagrams as well as the weathering classification presented in Table 2, the obtained F.S can be decreased by a suitable percentage under the relevant impact factor. This decrease indicates changes in shear strength at the relevant weathering stage. Regarding Figs. 9 and 10, which show the stability chart for various types of the marly slope, it was determined that limey marl, marly lime, and marls, are classified according to their carbonate-clay content. The charts estimate the F.S based on the geometry of the slope, the degree of weathering, and the geo unit compositions. Based on the stability chats, it can be stated that limey marl is more stable than marly lime or marls.

## Conclusions

The application of empirical classifications to provide engineering solutions for slope stabilization has a long background. Regarding the different purposes, various classifications were developed and implemented by specific essentials such as slope geometry, discontinuity network, seepage condition, etc. Although these classifications are used for different ranges of rocks, they are always erroneous for weak (clay-based) rocks. The presented study introduces the graphical stability charts for assessing the stability of clay-based weak rocks, including marlstones, lime marls, and marly limes. On the basis of extensive field surveys, geomechanical recording, sampling, and geotechnical experiments, the proposed LEM-based method is established. The method attempted to account for geotechnical and geological characteristics in order to provide an accurate estimate of the instability condition on slopes composed of weak/soft materials such as marls. Forty distinct slopes are evaluated for this study in the South Pars special zone (Assalouyeh), in southwestern Iran. The investigation's findings resulted in the creation of numerous charts to describe stability conditions in relation to geotechnical and geological conditions, particularly weathering. Utilizing these charts aid in understanding the current condition of slopes and obtaining several geotechnical requirements for stabilizations. According to the obtained results, a direct relationship has been obtained between weathering degree, angle of response, and shear strength of marls. This connection is used to prepare stability charts with different conditions for marly slope. The LEM methodology is used to estimate the F.S. in stability charts that are used to fast decisions in early stage of stability assessments. Charts provide information about F.S based on slope angle and slope height regarding weathering degree and geo-materials type (geo units). Using the geo units helps to properly understand the stability variation for marls. Based on the stability charts, it can be stated that limey marl is more stable than marly lime or marls.
